# Real-Time Correction and Long-Term Drift Compensation in MOS Gas Sensor Arrays Using Iterative Random Forests and Incremental Domain-Adversarial Networks

**DOI:** 10.3390/mi16090991

**Published:** 2025-08-29

**Authors:** Xiaorui Dong, Shijing Han

**Affiliations:** 1 School of Big Data and Basic Sciences, Shandong Institute of Petroleum and Chemical Technology, Dongying 257000, China; dongxr@sdipct.edu.cn; 2 School of Natural Resources and Surveying, Nanning Normal University, Nanning 530001, China

**Keywords:** sensor arrays, long-term drift compensation, real-time data correction, incremental domain-adversarial network, random forest, online learning

## Abstract

Sensor arrays serve a crucial role in various fields such as environmental monitoring, industrial process control, and medical diagnostics, yet their reliability remains challenged by sensor drift and noise contamination. This study presents a novel framework for real-time data error correction and long-term drift compensation utilizing an iterative random forest-based error correction algorithm paired with an Incremental Domain-Adversarial Network (IDAN). The iterative random forest algorithm leverages the collective data from multiple sensor channels to identify and rectify abnormal sensor responses in real time. The IDAN integrates domain-adversarial learning principles with an incremental adaptation mechanism to effectively manage temporal variations in sensor data. Experiments utilizing the metal oxide semiconductor gas sensor array drift dataset demonstrate that the combination of these approaches significantly enhances data integrity and operational efficiency, achieving a robust and good accuracy even in the presence of severe drift. This study underscores the efficacy of integrating advanced artificial intelligence techniques for the ongoing evolution of sensor array technology, paving the way for enhanced monitoring systems capable of sustaining high levels of performance over extended time periods.

## 1. Introduction

Sensor arrays have become indispensable in a variety of critical sectors, including environmental monitoring, industrial process control, medical diagnostics, and food safety assurance [1]. These systems offer the capability to acquire multi-dimensional and high-throughput data, enabling real-time detection and quantitative analysis of physical, chemical, or biological parameters in complex environments [2,3]. Despite their widespread adoption and promising potential, the reliability and long-term stability of sensor arrays remain a significant challenge in practical applications. One of the most pervasive issues encountered in sensor array deployments is sensor drift, a phenomenon characterized by the gradual, systematic deviation of sensor responses from their original calibrated baseline over time [4,5]. Sensor drift can result from various factors, including sensor aging, material degradation, fouling by sample matrices, changes in environmental conditions, electronic component instability, and even manufacturing inconsistencies or batch-to-batch variability [6]. As a result, the sensor outputs may no longer accurately reflect the true properties of the measured samples as time progresses. In addition to drift, sensor arrays are also susceptible to noise contamination arising from environmental interference or intrinsic electronic noise [7]. The combined effects of drift and noise can severely impair measurement precision and consistency, leading to unreliable data, erroneous trend interpretation, and ultimately, faulty decision-making. Given the increasing reliance on sensor arrays for high-stakes decision-making and automated process control, it is both technologically and economically imperative to develop robust, adaptive approaches that can compensate for long-term drift and maintain data integrity throughout the lifespan of a sensor system [8].

Traditionally, drift compensation strategies for sensor arrays have relied on periodic manual recalibration and the application of statistical signal processing techniques, such as baseline correction [5,9], calibration curve updates [10,11], principal component analysis (PCA) [5], and outlier filtering [12]. While these approaches can provide partial suppression of drift and noise, especially in short-term or controlled environments, they are often inadequate for addressing the complex, nonlinear, and unpredictable patterns of drift observed in real-world, long-term deployments. Moreover, conventional calibration routines are typically labor-intensive, time-consuming, and may interrupt continuous sensor operation [13,14]. These limitations make traditional methods difficult to scale for automated systems and reduce their practicality in real-time, high-throughput, or cost-sensitive industrial scenarios.

With the recent advancements in artificial intelligence (AI), machine learning (ML) and deep learning (DL) approaches have opened up new possibilities for accurate and adaptive sensor data processing [15,16]. Many researchers have proposed AI-based techniques, such as support vector machines (SVM) [7], artificial neural networks (ANN) [8], and recurrent neural networks (RNN) [17], for sensor drift compensation, anomaly detection, and signal prediction. These data-driven methods can learn complex temporal dependencies and intrinsic relationships from historical data, thus providing more flexible and effective solutions for dynamic, real-world environments. Vergara’s seminal work introduced the Gas Sensor Array Drift (GSAD) dataset, which quickly became a pivotal benchmark for studying long-term sensor drift compensation, offering a comprehensive, publicly available testbed that provided a foundational machine learning-based solution and catalyzed advancements in developing robust methodologies to maintain sensor system reliability over extended operational periods [6]. We extended the research based on Vergara’s work by proposing a novel data preprocessing method and an inertial machine learning framework for drift compensation in sensor systems, achieving significant improvements in classification accuracy and extending sensor lifespan without additional sensor production costs [7]. Liang proposed a multibranch long short-term memory-attention ensemble classification network (MLAEC-Net) to address sensor drift in electronic noses, improving recognition accuracy and model generalization by integrating LSTM, attention mechanisms, and classifier ensembles [18]. Bhadola proposed a multiplex network framework for analyzing sensor array data, leveraging PCA and planar maximally filtered graphs (PMFG) to investigate the evolving topological properties and sensor drift effects across multiple gases over time [19].

In this study, we propose a real-time data error correction and prediction framework for long-term drift compensation in sensor arrays. On one hand, we developed an iterative random forest-based data error correction algorithm to automatically identify and correct abnormal or drifting sensor responses in real time. On the other hand, we propose an incremental domain-adversarial network, which integrates the principles of domain-adversarial learning with an incremental adaptation mechanism. The combined framework ensures robust data reliability and supports long-term, uninterrupted sensor operation.

Section 2 describes the sensor array dataset and outlines specific data defects caused by long-term deployment. Section 3 presents details of the iterative random forest data error correction method. Section 4 introduces the proposed prediction algorithm based on a deep learning network named Incremental Domain-Adversarial Network (IDAN) with an incremental adaptation mechanism. Section 5 reports comparative experimental results and analyses. Section 6 discusses the implications, advantages, and limitations of the proposed approach. Finally, Section 7 concludes this paper.

## 2. Dataset

The Gas Sensor Array Drift (GSAD) dataset represents a monumental milestone in the field, standing as the most important and uniquely suited dataset for the study and development of advanced algorithms specifically addressing long-term sensor drift compensation. It is, in essence, the definitive and practically sole viable choice for thoroughly investigating long-term drift phenomena and rigorously evaluating adaptive learning algorithms in electronic nose systems [6]. Compiled over a period exceeding three years at the University of California, San Diego, this dataset’s unparalleled extensive temporal coverage, systematic sampling protocol, and realistic operational conditions solidify its status as an ideal and indispensable platform for researchers aiming to evaluate the long-term reliability and adaptability of pattern recognition techniques in the face of sensor aging and environmental variability. Data acquisition was performed using an array composed of sixteen metal-oxide semiconductor (MOS) gas sensors, evenly distributed among four commercially available sensor models (TGS2600, TGS2602, TGS2610, and TGS2620) [20]. This configuration ensures not only redundancy but also diversity in gas response characteristics, thereby enhancing robustness in the recorded signals. During the experimental campaign, the sensor array was repeatedly exposed to six representative volatile organic compounds—ethanol, ethylene, ammonia, acetaldehyde, acetone, and toluene—each administered at multiple controlled concentration levels. The measurement protocol included strictly timed exposure and cleaning phases, which minimized confounding effects and maximized the reproducibility of the observations. Each data sample in the GSAD dataset consists of a 128-dimensional feature vector reflecting extensive feature extraction from raw sensor signals, with eight key features derived from dynamic response profiles [7], such as amplitude, response and recovery time, and other physically relevant metrics computed for every sensor channel; the 128-column features are shown in Table 1.

Label information corresponding to the type and concentration of the analyte is provided for each record, facilitating both classification and regression analyses. The GSAD dataset consists of 13,910 samples systematically organized into 10 distinct batches based on their acquisition period, with a detailed distribution provided in Table 2. Such batching captures the chronological progression of sensor drift, offering a realistic scenario for investigating time-dependent performance degradation and the efficacy of drift mitigation methods. The GSAD’s design and scope make it particularly valuable for research on transfer learning, domain adaptation, and online learning as applied to chemical sensing.

The GSAD dataset is presented as being automatically collected; however, our previous research [7] revealed data integrity issues within it, which we believe represents a pioneering comprehensive identification. These observed anomalies, including missing data, sign errors, misplaced decimal points, and outliers, are likely symptomatic of signal corruption by noise or transmission channel malfunctions. Subsequently, Dennler identified a latent property in the GSAD dataset that limits its suitability for gas classification studies, revealing that temporal clustering and residual drift in the baseline response can significantly distort classification results [21]. These problems may distort statistical characteristics, impair data reliability, and compromise the effectiveness of subsequent analysis or machine learning models. Despite these recognized limitations, the GSAD dataset, with its extensive application and comprehensive time span, remains an indispensable resource. Crucially, its inherent data integrity challenges and identified temporal characteristics provide a robust and realistic testbed. This unique context allows us to thoroughly validate and demonstrate the effectiveness and resilience of our real-time calibration and long-term drift compensation methods, particularly in addressing severe real-world data imperfections. Section 3 will thus visualize and address these data errors using advanced error-correction algorithms, highlighting their impact and facilitating significant improvements in data quality.

## 3. Real-Time Data Error Correction Method Based on Random Forest

Real-time correction of sensor drift and measurement error is critical in ensuring the reliability of long-term sensor array deployments. Traditional calibration techniques often fail to generalize across diverse operational conditions or are computationally expensive for online adaptation. To address these challenges, we propose an online random forest regression framework for real-time error correction in sensor arrays, which leverages the collective information from all sensor channels to compensate for drift and outliers dynamically and robustly. The correction framework operates in two fundamental stages: (1) model training and validation and (2) online error correction and prediction. The framework is systematically designed for each sensor channel, exploiting the multivariate interdependence of the array structure.

### 3.1. Model Training and Validation

#### 3.1.1. Polarity Correction

For each incoming batch, basic physical constraints, primarily sign correction based on channel grouping, are enforced to eliminate implausible negative readings and harmonize channel orientation. To account for inherent sensor polarity and installation configuration, a sign correction rule is applied as follows:

(1) $$x_{i,j}\leftarrow \left|x_{i,j}\right|\;\mathrm{or}\;-\left|x_{i,j}\right|,\mathrm{depending}\mathrm{on}\mathrm{channel}\mathrm{group},$$

If 1≤i%8≤5, the value is replaced by its absolute value, thereby ensuring that channels in this group only record non-negative values. Conversely, for channels where i%8=0 or 6≤i%8≤7, the negative absolute value is assigned, systematically maintaining their expected negative polarity. 

#### 3.1.2. Model Training

Let the sensor array contain N channels. As the GSAD dataset was introduced earlier, we directly include it in our experimental setup, where it consists of N=128 sensor channels (i.e., $i\in \left\{1,2,\ldots ,N\right\}$). Each channel corresponds to a distinct sensor in the array, capturing parallel observations of the sensing environment.

For each individual sensor channel i, a dedicated random forest regressor [22] is trained. The regressor for channel i takes as input the measurements from all other channels but excludes the target channel measurement itself from the predictor set. The model learns to infer or reconstruct the reference signal for channel i based on information from the rest of the array.

A dedicated random forest regressor is trained, using the remaining channels and auxiliary metadata (excluding identifiers and the target channel itself) as predictive features. The random forest learns the mapping

(2) $${\hat{f}}_{i}:X_{\backslash i}\to y_{i},$$

where $y_{i}$ represents the reference measurements (either ground truth or values corrected in the first iteration) for channel i, and $X_{\backslash i}$ denotes the matrix of features excluding channel i, the batch index, and other meta-variables.

The model for each channel i is optimized to minimize the expected mean squared error (MSE) [23] between predicted and reference values across all training instances. The objective function is

(3) $${\hat{f}}_{i}=\underset{f\in F}{\arg\min}Ε\left[{\left(y_{i,j}-f\left(X_{\backslash i,j}\right)\right)}^{2}\right],$$

where F is the function space corresponding to all possible random forest mappings, $y_{i,j}$ is the j-th reference of channel i, $X_{\backslash i,j}$ is the corresponding feature vector for the j-th sample for channel i excluding channel i, and $E\left[\cdot \right]$ is the expectation for the training dataset.

#### 3.1.3. Model Validation

The training was conducted on a selected calibration batch (batch = 1), with parallel instantiation of 128 random forest models ($n_{estimators}=100$), each validated using in-sample metrics such as the coefficient of determination (R^2^) [24] and MSE:

(4) $$MSE_{i}=\frac{1}{n}{\sum_{j=1}^{n}\left(y_{i,j}-{\hat{y}}_{i,j}\right)}^{2},$$

(5) $$R_{i}^{2}=1-\frac{{\sum_{j}\left(y_{i,j}-{\hat{y}}_{i,j}\right)}^{2}}{{\sum_{j}\left(y_{i,j}-{\bar{y}}_{i,j}\right)}^{2}},$$

where ${\hat{y}}_{i,j}$ is the predicted value and ${\bar{y}}_{i,j}$ is the mean of observed values for channel i. The model, performance metrics, and diagnostic results were persistently stored for subsequent deployment and analysis, ensuring full reproducibility of the modeling process. The performance of the models trained on different batches of data across eight dimensions is shown in Table 3. As shown in Table 3, the random forest model trained using batch 1 as the training set exhibited superior performance, while the models trained with batch 1–2 or batch 1–3 showed relatively poorer performance in the F2, F5, and F8 dimensions. A detailed analysis of these results is provided in Section 3.3.

### 3.2. Real-Time Error Correction

Upon deployment, the trained models were used for online correction of newly acquired sensor data. The process involved the following steps.

#### 3.2.1. Data Preprocessing

To maintain the robustness and reliability of the prediction process, the same sign correction and channel orientation harmonization approach described in Section 3.1.1 was systematically applied prior to model inference. Although the predictive models had been previously trained on data subjected to these physical constraints, unforeseen noise or anomalies in the input data could still compromise prediction accuracy or even lead to model degradation. By replicating the preprocessing steps from the training phase, model performance is safeguarded against the adverse effects of noise, sensor polarity errors, and installation inconsistencies, thereby enhancing the generalizability and stability of the deployed models.

#### 3.2.2. Error Prediction

For each sensor channel i, the corresponding random forest model ${\hat{f}}_{i}$ predicts the expected true value ${\hat{y}}_{i,j}$ given the current feature vector $X_{\backslash i,j}$:


(6) $${\hat{y}}_{i,j}={\hat{f}}_{i}\left(X_{\backslash i,j}\right),$$

Since the system was designed for online deployment, data was processed sequentially as it was generated by the sensors. As a result, at most time points, the feature matrix X consisted of only a single record, corresponding to the current observation $X_{j}$. That is, the input to each prediction step was the latest reading, rather than a batch of samples.

#### 3.2.3. Correction Decision Rule

A relative residual check was conducted to determine whether the observed value should be replaced. Specifically, for each nonzero observation

(7) $$r_{i,j}=\frac{\left|{\hat{y}}_{i,j}-x_{i,j}\right|}{\left|x_{i,j}\right|},$$

the measurement was deemed erroneous and substituted with the model prediction


(8) $$x_{i,j}^{corr}=\left\{\begin{array}{c} {\hat{y}}_{i,j}\;\;\mathrm{if}\;r_{i,j}>\theta \\ x_{i,j}\;\;\mathrm{otherwise} \end{array}\right.,$$

In this study, the threshold parameter θ was set to 0.05 during the experimental phase. This value signified that a deviation of up to 5% between the predicted value and the original value is considered acceptable. If the deviation exceeded this 5% threshold, it was deemed a significant difference, prompting a correction through replacement to address notable outliers or decimal point inaccuracies. It is important to note that this specific θ value was chosen empirically and was not determined based on statistical criteria. However, in practical applications, the selection of θ can be refined according to statistical considerations, and its value is also highly relevant to the specific problem at hand. For instance, if the numerical distribution exhibits significant volatility, a larger value for θ might be more appropriate. This refinement could potentially involve leveraging confidence intervals from historical data or optimizing based on residual distributions.

#### 3.2.4. Iterative Correction

The data correction process was executed in two consecutive rounds. The rationale for this iterative approach lies in the inherently interconnected nature of sensor arrays, wherein measurement error or drift in one channel not only affects its own reading but may also implicitly influence regression predictions for other channels via shared latent factors or correlated dynamics. In the first iteration, the random forest models were applied to identify and correct substantial deviations in each channel, utilizing the current values of the remaining channels as predictive features. However, since the input features themselves may still have contained residual anomalies, the predictions in this pass may not have fully eradicated all data defects. By performing a second iteration over the now partially corrected data matrix, the feature vectors supplied to each regressor were of improved fidelity, thereby enabling more accurate and consistent restoration in the subsequent pass. This cascading correction strategy ensures that mutual error propagation is constrained, and the correction is no longer limited by initially corrupted inputs, leading to superior overall data quality.

The error correction module was designed using 128 independent random forest models, each corresponding to a specific sensor channel. These models were trained offline, and the primary operation during actual deployment was inference. For each incoming data sample from a single sensor channel, the prediction produced by a trained random forest model involved traversing a set of $n_{estimators}$ decision trees. Each individual tree traversal exhibited a complexity proportional to the depth of the tree. In the approach proposed in this paper, we configured $n_{estimators}=100$, with the maximum tree depth calculated automatically during training. However, through empirical observations, we found that the maximum depth of the trees in the constructed models was 16. Consequently, the inference complexity for a single prediction on a single channel was approximately $O\left(n_{estimators}\times d_{max\_depth}\right)$, or $O\left(100\times 16\right)$.

The iterative correction process for each sensor channel involved two such inference steps, resulting in a computational complexity of $2\times O\left(100\times 16\right)$. This represents a relatively small constant operation count. Furthermore, the total computational cost of correcting a full array snapshot remained fixed and independent of the historical data volume. From an empirical standpoint, when all 128 models resided in memory, the observed time for correcting one complete sensor array data entry (i.e., data from all 128 channels) was approximately 1.56 milliseconds, enabling high-speed real-time operation. This parallel processing capability was highly efficient. However, even under severe memory constraints where the 128 models had to be loaded serially for each operation, a complete correction could still be accomplished in approximately 7.8 s, demonstrating a viable processing option. This performance level unequivocally confirms the real-time capability of the proposed error correction method.

### 3.3. Effect of Error Correction

**Figure 1:**
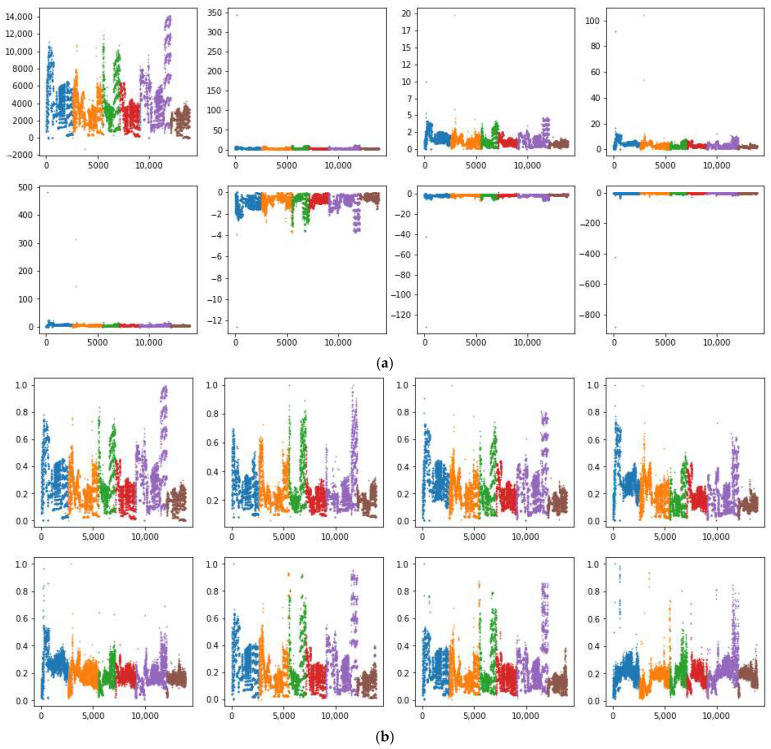

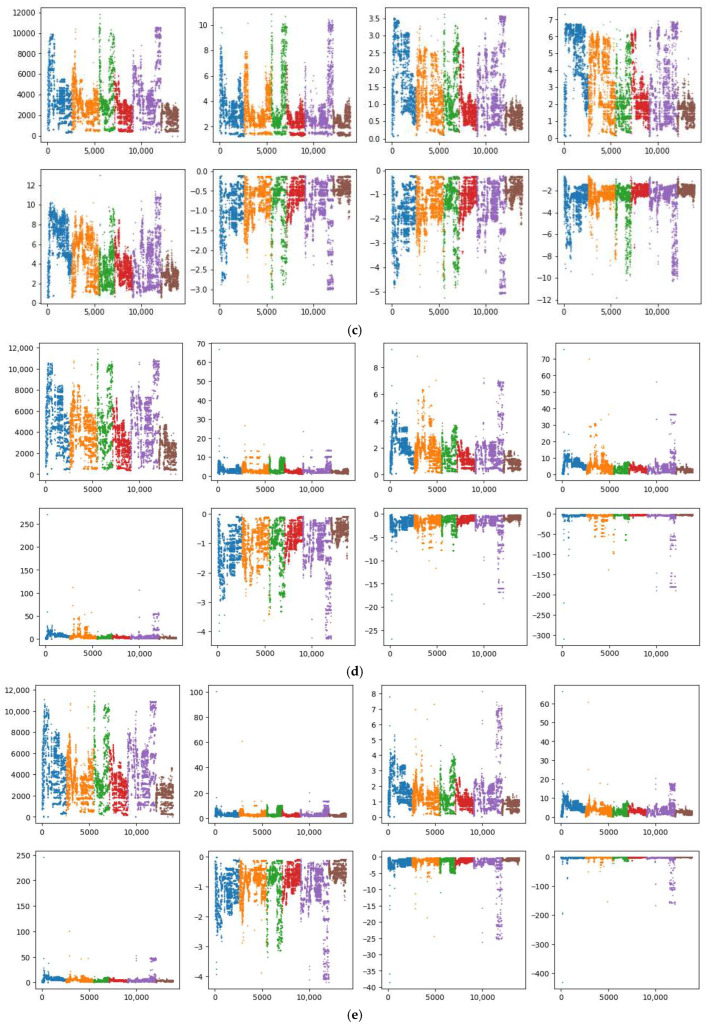
Results of the different processing methods on S5 sensor data, with different colors representing different gases: (**a**) raw data; (**b**) data distribution after processing with the method from ref. [12]; (**c**) data distribution after model correction using batch 1 data as the training set; (**d**) data distribution after model correction using batch 1–2 data as the training set; (**e**) data distribution after model correction using batch 1–3 data as the training set.

Compared with the method proposed in reference [7], the approach introduced in this study does not require researchers to manually fit empirical correction formulas, thereby offering a more objective and universal error correction framework. This method can be readily applied to both online and offline data calibration across a wide range of sensor types, significantly improving its generalizability and practical applicability. To visually illustrate the sensor drift behavior and how the proposed random forest correction method reshapes the data distribution, we compared the original data features with those of the corrected dataset. We trained random forest prediction models using batch 1, batch 1–2, and batch 1–3 as training sets, and subsequently compared the corrected results with both the raw sensor data and the outputs obtained using the methodology outlined in reference [7]. Taking the information collected by the S5 sensor as an example, the results after the five processing methods mentioned above are shown in Figure 1. Within each subplot of Figure 1, eight distinct scatter plots are shown, corresponding to feature values F1 through F8. The dataset is organized such that different gas types are first grouped and then arranged in chronological order, with each gas type identified by a unique color. In Figure 1, these colors specifically represent Ethanol, Ethylene, Ammonia, Acetaldehyde, Acetone, and Toluene. The horizontal axis is labeled as the sorted record index, and the vertical axis indicates the feature values.

As can be observed from Figure 1, substantial outliers in Figure 1a severely deteriorated the data distribution, where the use of MinMax normalization [25] compressed most values to zero, resulting in significant data distortion. In Figure 1b, the empirical correction method from reference [7] did alleviate some of these issues, especially in terms of reducing the overall number of outliers and better representing the actual data dispersions. Nonetheless, apparent problems persist in features F3, F4, F5, and F8, where the majority of values remain compressed below 0.5 or even 0.3, indicating that the data spread is still not optimal and considerable information loss occurs. Figure 1c, which illustrates the results of random forest-based correction using batch 1 as the training set, demonstrates a significantly improved data distribution. Here, the values for each feature are more evenly distributed, and the influence of outliers is notably mitigated. This reflects the strong learning and generalization capabilities of the random forest model under this configuration, allowing it to more effectively capture nonlinear relationships and compensate for the effects of noise or drift present in the raw data. However, a further increase in the training set, as shown in panel Figure 1d,e, did not yield better correction performance. In fact, the data quality declined considerably, as observed from the increasing data compression and the re-emergence of distributional distortions. The main reason can be attributed to the accumulation of noise and drift as more batches were included in the training set, which eventually hindered the model’s error correction capacity and may have even introduced additional bias.

Based on these observations, in the subsequent experiments, we selected the corrected dataset shown in Figure 1c—achieved by applying the random forest error correction model trained solely on batch 1 data—as the primary dataset for subsequent analysis and model development. This choice ensured an optimal balance between effective error correction and the retention of key data distribution characteristics.

## 4. Incremental Domain-Adversarial Network

**Figure 2:**
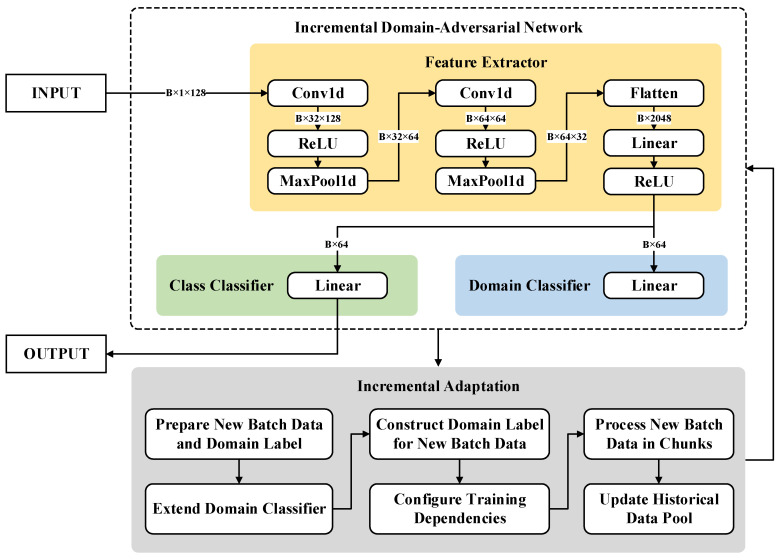
The network architecture and incremental adaptation mechanism of IDAN.

We propose an Incremental Domain-Adversarial Network (IDAN) which integrates the principles of domain-adversarial learning [26] with an incremental adaptation mechanism [27]. This approach addresses the need for efficient data error correction and drift mitigation by utilizing learning paradigms that generalize effectively over dynamic data distributions and can rapidly adapt to new domains with minimal retraining cost. The design of IDAN is depicted in Figure 2.

### 4.1. Network Architecture

The IDAN consists of three major modules:

1. Feature Extractor $G_{f}$. The feature extractor processes an input vector $x\in {\mathbb{R}}^{128}$ (to accommodate the sensor array) using a stack of convolutional and pooling layers, followed by a fully connected transformation to obtain a compact latent representation $h=G_{f}\left(x\right)\in {\mathbb{R}}^{64}$.
2. Label Predictor $G_{y}$. This branch, implemented as a fully connected layer, predicts the task class $\hat{y}=G_{y}\left(h\right)$, supporting multi-class classification ($y\in \left\{1,2.\ldots ,C\right\}$, with C=6 in this experiment).
3. Domain Classifier $G_{d}$. Also a fully connected layer, the domain classifier aims to infer from which domain or batch ($d\in \left\{1,2,\ldots ,K\right\}$) the feature embedding stems, promoting domain-invariant representation learning.

The full forward calculation can be summarized as

(9) $$h=G_{f}\left(x\right);\hat{y}=G_{y}\left(h\right);\hat{d}=G_{d}\left(h\right),$$

where $\hat{y}$, $\hat{d}$ denote the predicted outputs for class and domain, respectively.

### 4.2. Domain-Adversarial Training

To allow the feature extractor to learn domain-invariant embeddings, we employ domain-adversarial training by simultaneously minimizing the task classification loss $Loss_{class}$ and the domain classification loss $Loss_{domain}$:

(10) $$Loss_{class}=E_{\left(x,y\right)}\left[-\log P\left(y|x\right)\right],$$

(11) $$Loss_{domain}=E_{\left(x,d\right)}\left[-\log P\left(d|x\right)\right],$$

(12) $$Loss_{total}=Loss_{class}+\lambda Loss_{domain},$$

where λ is a hyperparameter balancing classification and domain-adversarial objectives (set to 1 in this study for simplicity). Both loss terms use the standard cross-entropy form.

### 4.3. Incremental Adaptation Mechanism

Standard domain-adversarial networks are trained jointly on data from multiple domains and are not inherently incremental. IDAN introduces an online adaptation mechanism as follows:

1. Initial Training. We train the network using labeled data from the first two sensor batches (domains). Each data sample is labeled as belonging to its batch for domain-discriminative learning. Let $D_{1}$, $D_{2}$ denote these batches.
2. Dynamic Domain Expansion. Upon the arrival of a new batch $D_{k+1}$, IDAN extends its domain classifier $G_{d}$ to accommodate the new domain by expanding the output layer and copying previous weights. Given a previously trained model with K domains, the domain classifier is extended to K+1 outputs; new weights for the additional domain are randomly initialized, while the existing ones are preserved to maintain prior knowledge. For a domain classifier with parameters $W_{d}\in {\mathbb{R}}^{K\times h}$, the extension is
  (13) $$W_{d}^{new}=\left[\begin{array}{c} W_{d}\\ w_{K+1} \end{array}\right],$$
  where $w_{K+1}\sim N\left(0,\sigma^{2}I\right)$.
3. Online Fine-Tuning. For each incoming batch, data is first normalized using parameters fitted from the initial training batches to ensure consistent feature scaling. The new batch is incrementally introduced in small chunks (buffer size is 50 in this experiment), mixed with a reservoir of historical samples (i.e., pool). For each chunk, IDAN is fine-tuned using combined current and historic batches, adapting to the new domain while retaining knowledge of previous ones. The combined sample update for each mini-batch can be formalized as
  (14) $$X_{train}={\left\{\left(x^{\left(i\right)},y^{\left(i\right)},d^{\left(i\right)}\right)\right\}}_{i\in Index_{hist}\cup Index_{chunk}},$$
  where $Index_{hist}$ denotes the set of indices for the samples selected from the historical data pool, and $Index_{chunk}$ denotes the set of indices for the samples in the currently incoming data chunk. This incremental learning continues for each subsequent batch, enabling real-time drift compensation as new domains emerge.

## 5. Experimental Comparison and Analysis

### 5.1. Experimental Datasets and Environment

For the purpose of investigating long-term sensor drift, two distinct experimental datasets based on the GSAD dataset, namely Dataset1 and Dataset2, were constructed based on specific partitioning rules:

1. Dataset1: Batch 1 is exclusively used as the training set, while batches k (where k = 2, 3, …, 10) are each used independently as the test set. This sequential evaluation is designed to assess the impact of temporal drift by examining the model’s predictive performance as the time gap between training and testing increases.
2. Dataset2: Batches 1 and 2 are jointly used to form the training set, and evaluation is conducted separately on batches k (k = 3, 4, …, 10). This approach aims to determine whether the inclusion of more initial data can mitigate the adverse effects of temporal drift and improve generalization to later, temporally distant batches.

Notably, due to the absence of toluene gas samples in batches 3 to 5, configurations such as using batches 1–3, 1–4, or 1–5 as the training set were not considered, as this would undermine the completeness and validity of the classification task. Additionally, the framework posits that training data stems from either laboratory-collected or manually calibrated samples; therefore, the use of extended historical data for training would not reflect practical application scenarios.

All experimental analyses were conducted within an Anaconda environment (Python 3.11, Individual Edition) on a platform equipped with an Intel i7-14700 3.40 GHz CPU and 64 GB RAM. The implementation leverages several third-party Python libraries, specifically NumPy (v1.26.4), Pandas (v2.0.3), SciPy (v1.16.0), Scikit-learn (v1.2.2), Matplotlib (v3.7.2), and Torch (v2.1.2+cu121) to ensure reproducibility and scalability of experimentation.

### 5.2. Experimental Results on Dataset1

To comprehensively evaluate the effectiveness of the proposed IDAN model, extensive experiments were conducted on Dataset1. We compared IDAN with several representative baseline methods, including Support Vector Machine (SVM) [6], Inertial-SVM (ISVM) [7], Convolutional Neural Network (CNN) [28], 1D Convolutional Neural Network (1DCNN) [29], TimesNet [30], and Long Short-Term Memory Network (LSTM) [31]. For all comparative experiments, the same data partitioning scheme was adopted as described in Section 5.1, with batch 1 used for model training and batches 2–10 employed for independent testing.

The primary evaluation metric employed in this study is classification accuracy (ACC), which provides a clear comparison of different models’ abilities to handle sensor drift over time. Table 4 summarizes the ACC results for all methods across the different test batches.

**Figure 3:**
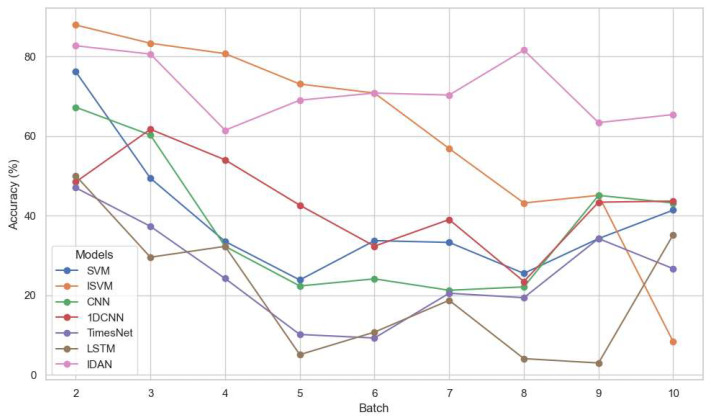
Experimental results for different methods on Dataset1.

To better visualize the influence of sensor drift and the temporal trend in model performance, we also present the ACC results in Figure 3. The figure illustrates, for each test batch, the accuracy trajectories of IDAN and all comparator models. It is evident that the performance gap widens over later batches, underscoring the effectiveness and robustness of IDAN in mitigating the adverse effects of long-term drift.

### 5.3. Experimental Results on Dataset2

**Figure 4:**
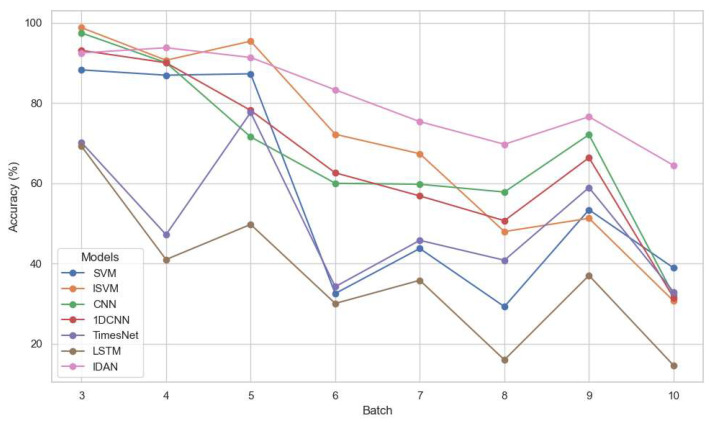
Experimental results for different methods on Dataset2.

In addition, we conducted a series of experiments on Dataset2, in which both batches 1 and 2 were merged to form a training set, while each of batches 3–10 was used as a separate test set. The same set of comparison methods (SVM, ISVM, CNN, 1DCNN, TimesNet, LSTM) was used, and the experimental settings remained identical to those described previously. Table 5 and Figure 4 present the experimental results on Dataset2.

### 5.4. Experimental Results Analysis

On Dataset1, which represents the scenario with the training set temporally furthest from most test batches, IDAN achieved an overall accuracy of 71.34%, surpassing the next-best baseline (ISVM, 52.07%) by more than 19 percentage points. While the other baseline methods exhibited significant accuracy decline as test batches shifted further in time, IDAN maintained a much steadier performance, highlighting its superior resilience to temporal drift. In test batches 7–10, IDAN consistently outperformed the second-best model by a margin of 14–35% in terms of accuracy. The performance of IDAN on Dataset1 is presented in Table 6, where the Precision (PR), Recall (RC), and F1-scores (F1) are computed using the “weighted” average rule.

On Dataset2, a similar pattern emerges. The inclusion of an additional training batch benefitted all models, yet IDAN continued to lead with the best overall accuracy of 76.29%. While traditional and deep learning baselines such as SVM and CNN improved notably on earlier test batches (e.g., batches 3–5), their results were unstable and dropped sharply in later sets (e.g., batch 10). In contrast, IDAN not only maintained high accuracy amidst severe drift, but also preserved robustness as the distribution shifted. Even during batches 7 to 10, the performance degradation remained minimal, demonstrating its adaptability to new patterns and enhanced generalization to distant temporal domains. The performance of IDAN on Dataset2 is shown in Table 7, with the performance metrics consistent with those reported in Table 6.

The traditional methods had inherent limitations in drift-prone environments. Classical models like SVM and ISVM displayed restricted resistance; deep learning approaches (CNN, 1DCNN, LSTM) tended to overfit to the training window and falter on evolved distributions. TimesNet, despite being tailored for time series, also lagged behind IDAN, likely due to insufficient modeling of complex, non-stationary drift typical in real-world sensor data. In addition, extending the training set (as in Dataset2) alleviated accuracy loss to some degree for all methods, confirming the value of expanded calibration. However, this positive effect soon plateaued and failed to fully address long-term drift, as reflected in the later test batch results. Consequently, IDAN’s edge became more prominent as the temporal gap widened, reinforcing its practicality for long-term monitoring tasks where frequent recalibration is impractical.

The superior performance of IDAN, particularly its sustained accuracy across later batches, is a direct consequence of its integrated domain-adversarial learning and incremental adaptation mechanisms. Unlike traditional models which struggle with shifting data distributions, the domain-adversarial component of IDAN explicitly forces the feature extractor to learn representations that are invariant to domain changes, effectively decoupling gas classification from temporal drift. Furthermore, the incremental adaptation mechanism allows IDAN to continuously update its knowledge with new data batches without catastrophic forgetting, a critical capability for maintaining performance over extended deployment periods.

## 6. Discussion

In terms of error correction, this study proposes a real-time data error correction method based on random forests, which is capable of effectively addressing measurement anomalies and compensating for sensor drift to a certain degree. This approach introduces an iterative framework that exploits the collective information from multiple sensor channels, thereby dynamically mitigating both drift and the presence of outliers. Moreover, the two-stage correction process continuously refines the predictions by utilizing inter-channel complementarity, thus reducing the propagation of errors across the sensor network.

In terms of drift compensation and online prediction, while existing strategies, such as SVM and more conventional deep learning architectures, have shown merit within static environments, they often struggle to maintain high performance levels when faced with the complexities and variabilities of long-term drift. Methods such as ISVM yield improved results compared to SVM, yet still fall short of the robustness and accuracy afforded by IDAN. The proposed IDAN framework represents a significant advancement in sensor drift compensation. Its ability to achieve robust and high accuracy under severe and long-term drift conditions addresses a major bottleneck in deploying reliable sensor arrays for critical applications. By simultaneously optimizing both classification and domain discrimination tasks, the IDAN ensures that learned representations are robust to variations often observed in real-world settings. The iterative fine-tuning process inherent in IDAN serves to bolster its resilience against drift. This iterative approach, implemented through an online adaptation mechanism, enables the model to continually update its learned parameters and maintain relevance as new data batches are introduced. This not only reduces the need for costly and time-consuming manual recalibration but also enables the development of truly autonomous and self-adapting sensor systems. The combination of real-time error correction and continuous drift compensation through incremental domain adaptation is especially crucial for industrial and environmental monitoring where sensor data is constantly evolving and immediate, accurate insights are required.

Despite the promising outcomes presented, it is crucial to acknowledge some limitations inherent to our current framework. The reliance on periodic adjustments via model retraining imposes a burden on computational resources, particularly in scenarios involving extensive datasets or limited processing capabilities. Future work may focus on optimizing the error correction algorithm and exploring more sophisticated unsupervised or semi-supervised learning techniques that can balance computational load with model fidelity.

## 7. Conclusions

This study demonstrates the effectiveness of real-time data error correction and prediction with online learning for long-term drift compensation in sensor arrays through the application of IDAN. By integrating domain-adversarial learning with an iterative error correction approach, IDAN not only outperforms traditional methods in handling sensor drift but also remains adaptable in the face of evolving measurement conditions. This dual capability ensures sustained data integrity and reliability over extended deployment periods, making it an essential model for contemporary sensor applications. Furthermore, while the current study focuses primarily on gas sensor arrays, the principles underlying the IDAN, coupled with real-time data error correction, can be extended to other sensor types, including temperature and humidity sensors. Future explorations can examine the applicability of our framework across multi-modal sensor systems, potentially enriching the robustness and accuracy of data across a diverse spectrum of sensing technologies.

## Figures and Tables

**Table 1:** The 128-column features of the GSAD dataset.

Feature Type Abbr.	Features (S1)	Features (S2)	Features (S3)	…	Features (S16)
F1	1.	9.	17.	…	121.
	ΔR_S1	ΔR_S2	ΔR_S3		ΔR_S16
F2	2.	10.	18.	…	122.
	$\left\|\Delta R\right\|\_S1$	$\left\|\Delta R\right\|\_S2$	$\left\|\Delta R\right\|\_S3$		$\left\|\Delta R\right\|\_S16$
F3	3.	11.	19.	…	123.
	$ema_{0.001}I\_S1$	$ema_{0.001}I\_S2$	$ema_{0.001}I\_S3$		$ema_{0.001}I\_S16$
F4	4.	12.	20.	…	124.
	$ema_{0.01}I\_S1$	$ema_{0.01}I\_S2$	$ema_{0.01}I\_S3$		$ema_{0.01}I\_S16$
F5	5.	13.	21.	…	125.
	$ema_{0.1}I\_S1$	$ema_{0.1}I\_S2$	$ema_{0.1}I\_S3$		$ema_{0.1}I\_S16$
F6	6.	14.	22.	…	126.
	$ema_{0.001}D\_S1$	$ema_{0.001}D\_S2$	$ema_{0.001}D\_S3$		$ema_{0.001}D\_S16$
F7	7.	15.	23.	…	127.
	$ema_{0.01}D\_S1$	$ema_{0.01}D\_S2$	$ema_{0.01}D\_S3$		$ema_{0.01}D\_S16$
F8	8.	16.	24.	…	128.
	$ema_{0.1}D\_S1$	$ema_{0.1}D\_S2$	$ema_{0.1}D\_S3$		$ema_{0.1}D\_S16$

**Table 2:** Record distribution for each gas in different batches.

Batch Id	Month Ids	Total Number	The Number of Records for Each Gas in Each Batch					
			Ethanol	Ethylene	Ammonia	Acetaldehyde	Acetone	Toluene
1	1, 2	445	90	98	83	30	70	74
2	3, 4, 8, 9, 10	1244	164	334	100	109	532	5
3	11, 12, 13	1586	365	490	216	240	275	0
4	14, 15	161	64	43	12	30	12	0
5	16	197	28	40	20	46	63	0
6	17, 18, 19, 20	2300	514	574	110	29	606	467
7	21	3613	649	662	360	744	630	568
8	22, 23	294	30	30	40	33	143	18
9	24, 30	470	61	55	100	75	78	101
10	36	3600	600	600	600	600	600	600
**Total Number**		13,910	2565	2926	1641	1936	3009	1833

**Table 3:** The performance of the models trained on different batches of data across eight dimensions.

Train Set	Dimension	Min_MSE	Max_MSE	MSE	R^2^
batch 1	F1	3,863,654	1824	685,523	0.9995
	F2	0.0728	0.0026	0.0151	0.9990
	F3	0.5913	0.0004	0.0904	0.9990
	F4	0.7696	0.0039	0.1738	0.9984
	F5	22.6928	0.0400	3.8387	0.9863
	F6	0.2083	0.0003	0.0354	0.9994
	F7	2.6754	0.0012	0.3308	0.9989
	F8	48.4364	0.0656	7.6114	0.9816
batch 1–2	F1	1,652,796	1580	324,721	0.9995
	F2	117,057	0.0007	9881	0.9278
	F3	0.9593	0.0004	0.1241	0.9926
	F4	20.6208	0.0346	3.6756	0.9787
	F5	4970.1092	1.4989	417.5656	0.9532
	F6	0.1426	0.0001	0.0337	0.9969
	F7	7.4404	0.0240	1.2041	0.9736
	F8	403.4141	7.4268	45.2815	0.9253
batch 1–3	F1	804,049	1257	174,602	0.9997
	F2	56,162	0.0006	4700.3703	0.9383
	F3	0.6775	0.0002	0.0799	0.9945
	F4	11.4853	0.0212	2.0481	0.9865
	F5	3608.4700	0.7870	297.3978	0.9580
	F6	0.0931	0.0001	0.0210	0.9985
	F7	2.7795	0.0113	0.4674	0.9801
	F8	142.3704	2.4981	19.1085	0.9470

**Table 4:** Experimental results for all methods across the different test batches on Dataset1.

Models	Overall ACC(%)	ACC (%) of Test Set Batch								
		2	3	4	5	6	7	8	9	10
SVM	41.13	76.21	49.43	33.54	23.85	33.73	33.29	25.51	34.25	41.41
ISVM	52.07	87.94	83.35	80.75	73.10	70.83	56.88	43.20	45.11	8.31
CNN	37.46	67.28	60.28	32.30	22.34	24.13	21.26	22.11	45.11	43.19
1DCNN	42.72	48.47	61.73	54.04	42.64	32.35	39.03	23.47	43.40	43.67
TimesNet	25.02	47.03	37.33	24.22	10.15	9.26	20.51	19.39	34.26	26.69
LSTM	25.05	50.08	29.57	32.30	5.08	10.74	18.74	4.08	2.98	35.25
IDAN	71.34	82.72	80.64	61.49	69.04	70.83	70.33	81.63	63.40	65.42

**Table 5:** Experimental results for all methods across the different test batches on Dataset2.

Models	Overall ACC(%)	ACC (%) of Test Set Batch							
		3	4	5	6	7	8	9	10
SVM	47.28	88.27	86.95	87.3	32.52	43.75	29.25	53.4	38.91
ISVM	61.22	98.8	90.68	95.43	72.22	67.37	47.96	51.28	30.67
CNN	57.58	97.48	90.06	71.57	60.0	59.76	57.82	72.13	32.14
1DCNN	56.17	93.13	90.06	78.17	62.61	56.85	50.68	66.38	31.50
TimesNet	43.90	70.18	47.20	77.66	34.22	45.78	40.82	58.94	32.92
LSTM	32.68	69.23	40.99	49.75	30.04	35.82	15.99	37.02	14.61
IDAN	76.29	92.50	93.79	91.37	83.30	75.37	69.73	76.60	64.47

**Table 6:** The performance of IDAN on Dataset1.

Test Set Batch	ACC (%)	PR (%)	RC (%)	F1 (%)
2	82.72	84.95	82.72	81.97
3	80.64	84.55	80.64	80.27
4	61.49	70.03	61.49	58.62
5	69.04	61.99	69.04	62.56
6	70.83	94.79	70.83	73.11
7	70.33	81.04	70.33	66.07
8	81.63	83.98	81.63	81.00
9	63.40	54.48	63.40	56.24
10	65.42	75.54	65.42	65.91

**Table 7:** The performance of IDAN on Dataset2.

Test Set Batch	ACC (%)	PR (%)	RC (%)	F1 (%)
3	92.50	93.95	92.50	92.50
4	93.79	93.87	93.79	93.73
5	91.37	92.57	91.37	91.28
6	83.3	97.41	83.30	87.40
7	75.37	86.27	75.37	71.63
8	69.39	80.67	69.39	71.39
9	76.60	68.84	76.60	70.70
10	64.47	67.77	64.47	64.88

## Data Availability

The dataset and codes reported in this study are publicly available in the GitHub repository at https://github.com/dongxr2/DriftComp-IDAN/ (accessed on 28 July 2025).
